# Confined bioprinting and culture in inflatable bioreactor for the sterile bioproduction of tissues and organs

**DOI:** 10.1038/s41598-024-60382-2

**Published:** 2024-05-14

**Authors:** Alexandre Dufour, Lucie Essayan, Céline Thomann, Emma Petiot, Isabelle Gay, Magali Barbaroux, Christophe Marquette

**Affiliations:** 1 3d.FAB, CNRS, INSA, CPE-Lyon, UMR5246, ICBMS, Universite Claude Bernard Lyon 1, Villeurbanne, France; 2 Sartorius Stedim FMT, Aubagne, France

**Keywords:** Bioprinting, Bioreactor, Confined environment, Organs production, Tissue engineering, Regenerative medicine

## Abstract

The future of organ and tissue biofabrication strongly relies on 3D bioprinting technologies. However, maintaining sterility remains a critical issue regardless of the technology used. This challenge becomes even more pronounced when the volume of bioprinted objects approaches organ dimensions. Here, we introduce a novel device called the Flexible Unique Generator Unit (FUGU), which is a unique combination of flexible silicone membranes and solid components made of stainless steel. Alternatively, the solid components can also be made of 3D printed medical-grade polycarbonate. The FUGU is designed to support micro-extrusion needle insertion and removal, internal volume adjustment, and fluid management. The FUGU was assessed in various environments, ranging from custom-built basic cartesian to sophisticated 6-axis robotic arm bioprinters, demonstrating its compatibility, flexibility, and universality across different bioprinting platforms. Sterility assays conducted under various infection scenarios highlight the FUGU’s ability to physically protect the internal volume against contaminations, thereby ensuring the integrity of the bioprinted constructs. The FUGU also enabled bioprinting and cultivation of a 14.5 cm^3^ human colorectal cancer tissue model within a completely confined and sterile environment, while allowing for the exchange of gases with the external environment. This FUGU system represents a significant advancement in 3D bioprinting and biofabrication, paving the path toward the sterile production of implantable tissues and organs.

## Introduction

The future of organ and tissue biofabrication heavily depends on 3D bioprinting technologies^1^ and their capacity to produce sterile, mature, functional, and physiological-sized tissues. Regardless of the bioprinting technology (i.e. laser-assisted, ink-jet, micro-extrusion or volumetric bioprinting), maintaining sterility is a critical issue^2,3^. Today, for 60% of low-cost bioprinters and 67% of high-end bioprinters, manufacturers and users rely on placing the bioprinters within a biosafety cabinet (BSC) to address sterility. However, a biosafety cabinet is not sterile, but it is aseptic^4^. Sterility, as defined by the Centers of Disease Control and prevention (CDC), refers to “state of being free from all living microorganisms”. Achieving sterility typically involves processes such as autoclaving, filtration, or irradiation to eliminate all microbial life. On the other hand, asepsis is defined as “prevention from contamination with microorganisms. Includes sterile conditions on tissues, on materials, and in rooms, as obtained by excluding, removing, or killing organisms”^5^. In a BSC, aseptic techniques are employed to maintain a sterile environment within the cabinet by minimizing the introduction of contaminants. Maintaining absolute sterility within a BSC is very challenging due to the continuous airflow and potential for introduction of contaminants from the surrounding environment.

In BSC and remaining bioprinters with a built-in “sterilization system” ^4^, ultraviolet (UV) and HEPA air filters are used to kill and removed microorganisms. However, ultraviolet radiation has long been recognized as a misleading and misused process for routine sterilization within BSCs. While the bactericidal properties of UV radiation have been acknowledged for a century, its efficacy within BSCs—where bacterial contaminants are often dry and protein-covered—has been deemed unreliable, even after extended exposure periods^6^. Consequently, the ability of such systems to maintain sterile conditions within apparatuses of complex geometries and fittings like bioprinters is questionable.

Culturing bioprinted tissues is also a huge challenge when their size approaches organ dimension (at least 100 cm^3^), since culture containers currently used for small bioprinted tissues (i.e. Petri dish, 6- or 12-well plate) are useless. The most advanced strategies to cope with this problem are the design of specific bioreactor-like culture vessels^7,8^. These systems are mainly polymeric air-tight devices in which pH and oxygen controls are maintained through perfusion of culture medium. Only a few bioreactor-based start-up companies specializing in engineering patient-specific tissue grafts have emerged, with a clear unmet need for integrating bioreactor devices with 3D bioprinting^9^.

Regarding sterility, these plastic bioreactors are partially effective since during the transfer to cultivation vessels the tissues are exposed to a non-sterile environment, which poses a significant risk of contaminating the bioprinted tissues/organs.

Consider if it were possible to both produce and cultivate 3D tissues within a completely confined and sterile environment, having the capacity to exchange freely gases with an external environment. To achieve this, the entire biofabrication process, including bioink preparation, bioprinting, cultivation, and stimulation, would need to occur within a sealed gas-permeable sterile container. This implies a unique challenge in bioprinting: introducing the bioprinting tool into this container and allowing it to move in three dimensions without compromising sterility. Moreover, this step is just the beginning; the vessel must also facilitate other standard cultivation and tissue qualification steps like nutrient’s feeding thanks to medium perfusion and monitoring of strategic culture parameters and tissue functions, all within the same sealed environment. An additional really interesting feature might be the capacity to vary, in real-time, the container's internal volume so that culture medium availability can be finely tuned for example.

We have addressed all these challenges by developing an advanced biofabrication platform enabling both bioprinting and cultivation of 3D tissues within a confined and sterile space.

## Conceptual approach

**Figure 1:**
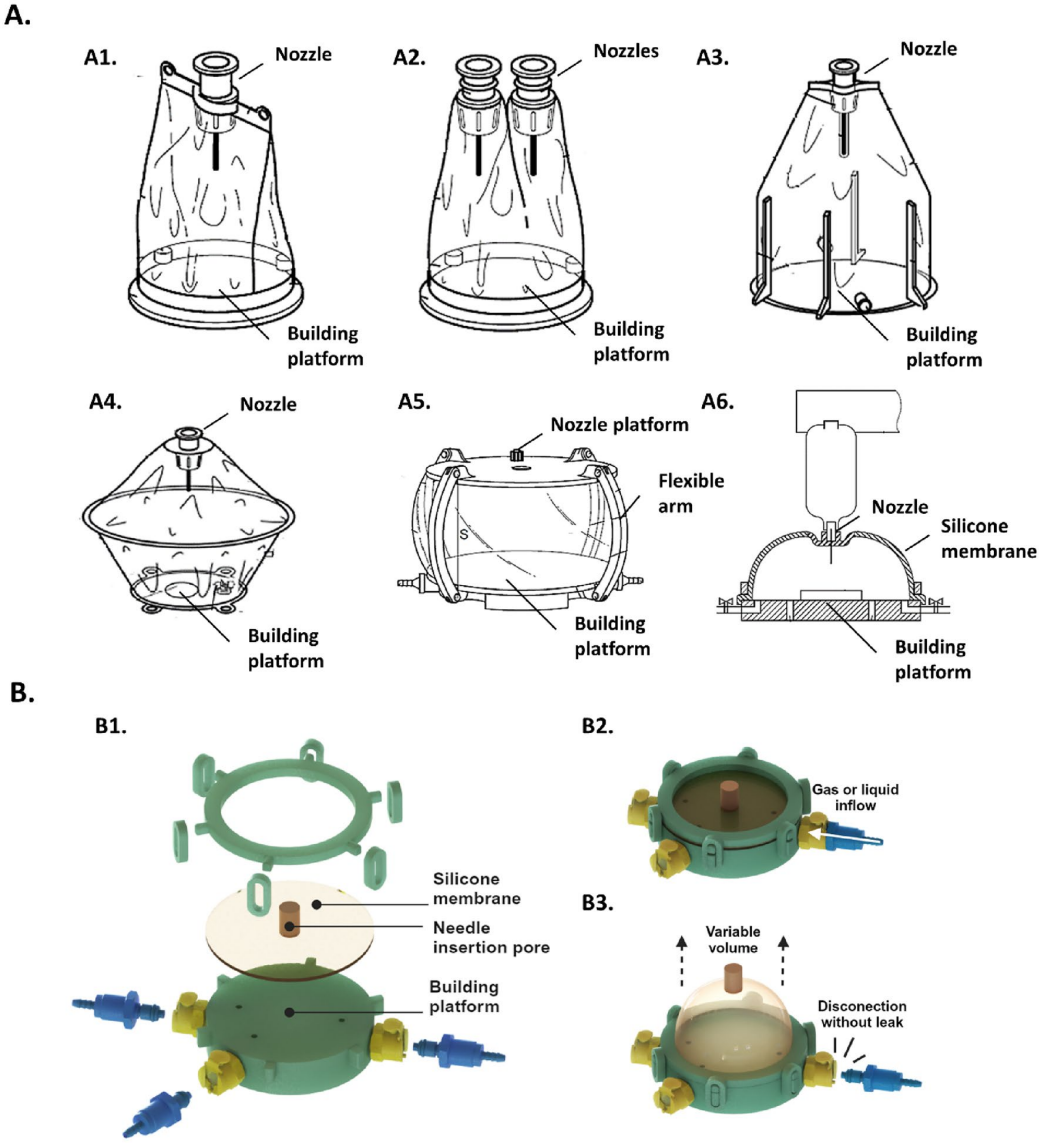
Conceptual iterations leading to the development of the FUGU design. (**A**) Multiple concepts were evaluated. A1/ single nozzle printing bag concept. A2/ multiple nozzle printing bag concept. A3/ Fully compliant printing bag concept wherein side wall integrates compliant solid material. A4/ Double printing bag concept. A5/ Flexible wall printing box concept. A6/ Inflatable confined printing chamber concept (i.e. FUGU). (**B**) Detailed view of the FUGU components. B1/ Split view of the different components of the FUGU. B2/ View of the assembled FUGU. B3/ Illustration of the inflatable membrane and the leak-free connection.

Our advanced biofabrication platform was developed through several iterations involving various materials and designs summarized in Fig. 1. These iterations included: (1) a commercially available flexible bag integrated with one or more bioprinting nozzles and bound to a building platform (Fig. 1-A1 and A2); (2) a flexible wall printing box concept composed of a nozzle platform and a building platform linked by a flexible silicone membrane and supported by flexible arms (Fig. 1-A5); (3) a hybrid of the first two designs (Fig. 1-A3 and A4). The final iteration resulted in an inflatable biofabrication platform comprising a silicone membrane attached to a building platform equipped with fluid management, as depicted in Fig. 1-A6. This design, named The Flexible Unique Generator Unit (FUGU), was recognized as the most comprehensive confined system, allowing complete flexibility during bioprinting (Fig. 1-B), adjusting internal culture volume, managing the liquid culture phase, enabling direct observation through the transparent silicone membrane, and facilitating gas exchange thanks to gas permeability of the silicone membrane^10^.

After defining the FUGU concept and developing a prototype, its operational functions and characteristics were thoroughly evaluated and documented. The present report details the selection of materials for all FUGU components, their fabrication steps and architecture, the integration of 3D bioprinting, sterility tests, tissue culture assessments and gas permeation.

## Results and discussion

### Conception and production of FUGU prototypes

The FUGU system is an intricate assembly of several components, each manufactured independently and showcased in Fig. 1-B. Key elements include, and will be discussed further in the following section:

1. Flexible Silicone Elastomeric Membrane: A 1 shore A Liquid Silicone Rubber (LSR) silicone elastomeric moulded membrane.
2. Needle Insertion Pore: This is 3D printed using a 70 shore A LSR. The dimensions of the part are 15 mm in height, 1 mm in outer diameter, and 7 mm in inner diameter.
3. Solid Bioprinting Platform, Membrane Holder, and Clamps: Initially, these parts were machined from stainless steel 316/316L. For disposability and compliance with single-use strategy in the bioprocess field^11^, we explored their production through 3D printing using medical-grade polycarbonate (MAKROLON®). The building platform surface has to be perfectly flat and smooth, to be compatible with initial bioprinter Z-axis calibration. The dimensions of the platform are 19 mm in height and 40 mm in outer diameter. The dimensions of the membrane holder are 5 mm in height, 42 mm in outer diameter, and 32 mm in inner diameter. The dimensions of the clamps are 11 mm in length, 4.5 mm in width, and 22.5 mm in height for the outer dimensions, and 5 mm in length, 4.5 mm in width, and 16.5 mm in height for the inner dimensions.
4. Fluidic Operations Ports: To aid fluid operations within the system, valved connectors were integrated directly into the printing bed.

The assembled FUGU device underwent steam sterilization. All components (both flexible and solid) and materials (silicone, stainless steel, MAKROLON® polycarbonate) of the FUGU device were able to withstand steam sterilization without compromising the geometry of the parts. However, the durability of the silicone membrane and MAKROLON® polycarbonate regarding this sterilization process was not assessed, as they are intended for single-use.

**Figure 2:**
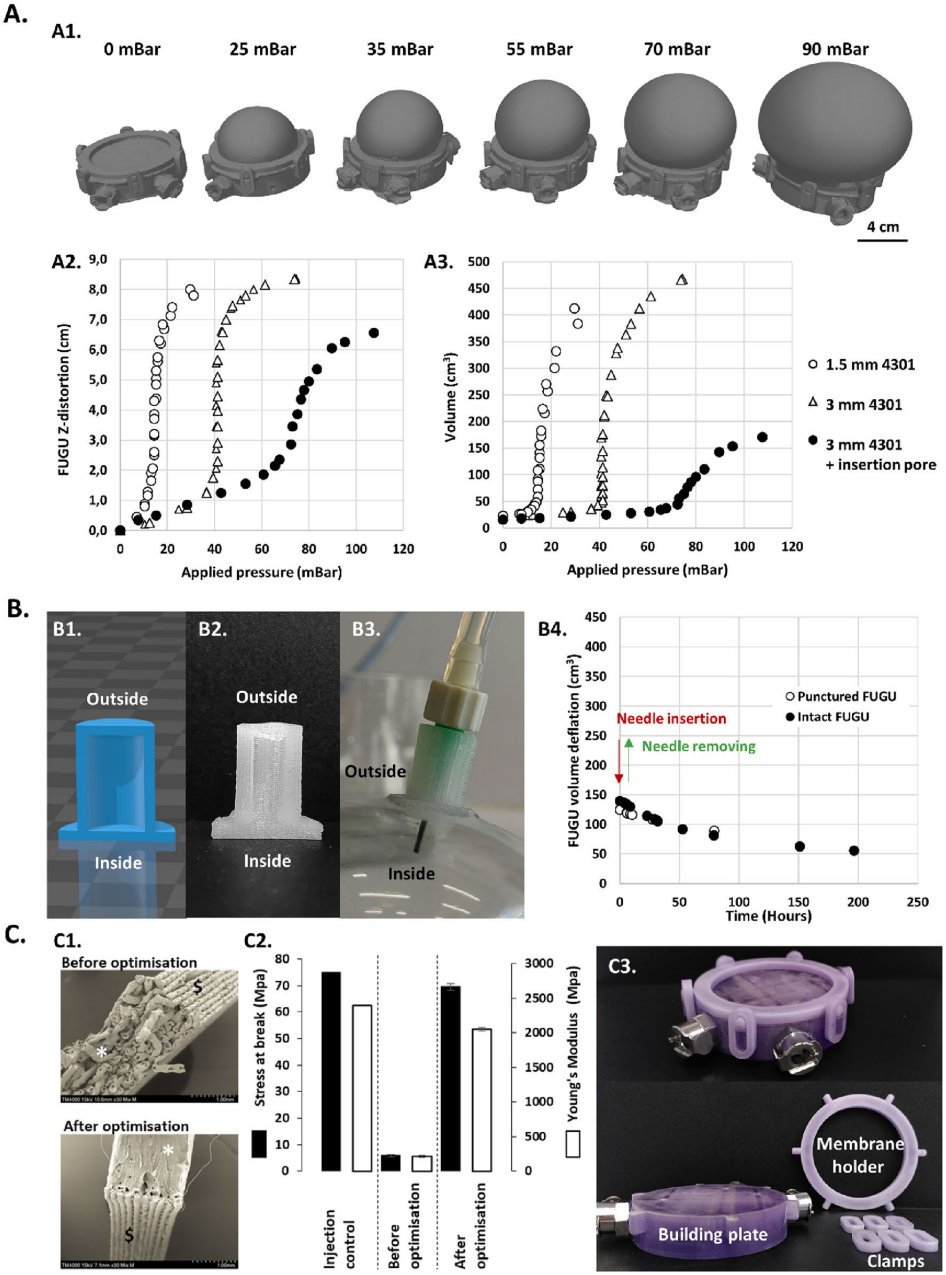
FUGU prototyping and characterization. (**A**) Characterisation of the inflation of the FUGU silicone membrane. A1/ 3D scans of a 1.5 mm thick FUGU membrane while it was being inflated using increasing pressure. A2/ Z-distortion of 3 FUGU silicone membranes of varying thicknesses according to the applied inflation pressure. A3/ Calculated (using 3D scan) internal volume of the FUGU according to the applied inflation pressure (data extracted from 3D scans). (**B**) Design and characterization of the FUGU insertion pore. B1/ 3D model cross-section of the pore. B2/Cross-section view of the 3D printed insertion pore. B3/ Bioprinting needle (1.6 cm long, 800 µm diameter) inserted into the insertion pore. B4/ Puncture test of the FUGU insertion pore. The arrows represent the insertion and removal of the needle in the case of the puncture FUGU. (**C**) Building plate production using 3D printed MAKROLON® polycarbonate on the FreeFormer 300-3X technology. C1/ Scanning electron microscopy images of MAKROLON®test parts cross-section, before and after printing optimisation. C2/ Mechanical characterization of the 3D printed MAKROLON® parts and comparison with the moulded reference. C3/ Images of the final FUGU MAKROLON® parts.

The inflatable silicone elastomeric membrane and its insertion pore are the corner stone of the FUGU system. These components ensure system compliance during 3D bioprinting and enable sterile needle insertion. Figure 2A demonstrates how homogeneous silicone membranes of varying thicknesses (1.5 and 3 mm) distort under different inflation pressures. Notably, thinner membranes require less pressure for comparable distortion, offering a broad range of internal volumes (from 0 to 460 mL, Fig. 2-A3). This variability allows for unprecedented adaptability in the internal size of the FUGU during bioprinting and culture. Of note, adding an insertion pore to the membrane had a similar effect to increasing the membrane thickness, resulting in a reduction in inflation capability.

The needle insertion pore itself serves three main functions: pierceability, sealing after puncture, and barrier to microbiological contamination. Its design, detailed in Fig. 2-B1, incorporates a 1 cm diameter tube with 2 mm thick walls, placed between two 2 mm thick diaphragms. The present configuration, produced using a specific 3D printable LSR silicone formulation^12^, ensures its functionality and integrity. Its 70 shore A composition ensure its geometrical stability while the FUGU membrane inflate (Fig. 2-B3).

The sealing property of the insertion pore was assessed by monitoring the FUGU's deflation rate before and after inserting an 800 µm diameter needle.

Regardless of whether the pore was pierced or not, an average deflation rate value of 0.4 cm^3^/hour was observed (Fig. 2-B4). This observation sheds light on two important behaviours: the punctured pore is efficiently sealed after removal of the needle, and the silicone membrane possesses a clear gas permeation. This last point is a key attribute for maintaining optimal oxygen and pH levels in a CO_2_ incubator during tissue cultivation. Indeed, pH is commonly maintained thanks to a buffering couple of dissolved CO_2_/bicarbonate. This permeability was evaluated using gas diffusion measurements through a 2 mm silicone membrane. The 1 shore A LSR silicone elastomeric membrane was found to have high permeability, with 177 ± 4 Barrer, 370 ± 2 Barrer and 1747 ± 20 Barrer, for N_2_, O_2_ and CO_2_, respectively.

Lastly, to achieve a fully disposable version of the FUGU system, all solid components- were 3D printed from MAKROLON®, a medical-grade polycarbonate, using the Freeformer technology. The optimized process yielded parts with mechanical properties closely matching those of injection-moulded MAKROLON®, as shown in Fig. 2-C1 and C2. Dense, homogeneous and watertight polycarbonate parts were obtained (Fig. 2-C1) with mechanical properties corresponding to 85% and 93% of the injected MAKROLON® Young’s modulus and stress at break, respectively (Fig. 2-C2).

### Evaluation of FUGU capacity as an advanced versatile biofabrication platform

**Figure 3:**
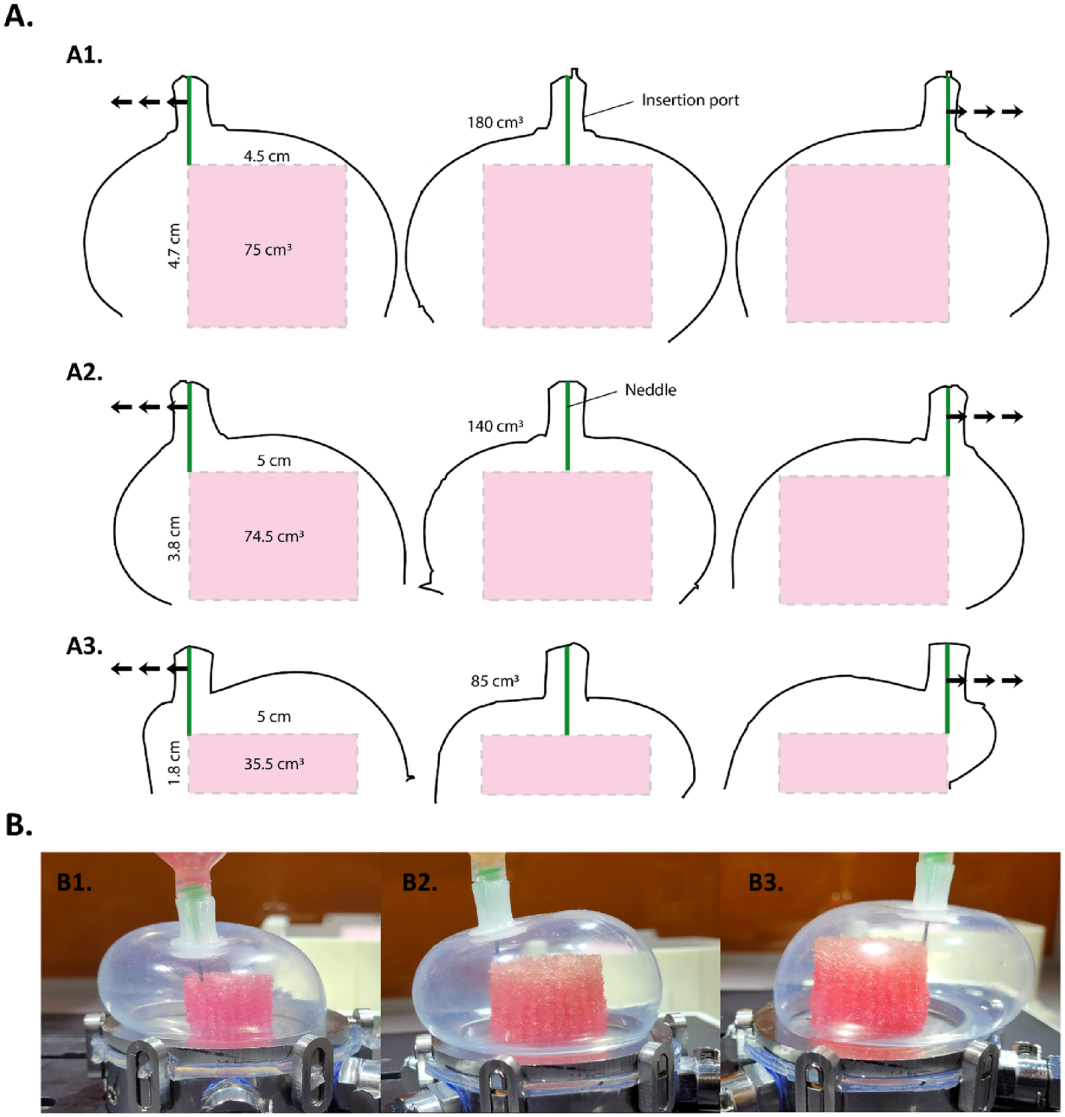
Range of motion and biopriting volumes in the FUGU device. (**A**) Deformation profiles extracted from live bioprinting pictures. A1/ Within a 180 cm^3^ inner volume FUGU. A2/ Within a 140 cm^3^ inner volume FUGU. A3/ Within an 85 cm^3^ inner volume FUGU. (**B**) Images of the live bioprinting within the FUGU. B1/ Within an 85 cm^3^ inner volume FUGU. B2/ Within a 140 cm^3^ inner volume FUGU. B3/ Within a 180 cm^3^ inner volume FUGU.

The aim of the advanced biofabrication platform was to allow for a wide range of geometries and sizes to be bioprinted in a sterile and confined environment. Thus, to describe and establish the boundaries of the bioprintable volume within the FUGU system, a detailed image analysis was conducted. Such analysis involved observing the movement of a standard micro-extrusion bioprinting needle within the FUGU system. To do so, a 1.6 cm long 800 µm diameter needle was inserted into the FUGU at various inflation volumes. Three different FUGU volumes (85, 140, and 180 cm^3^) were examined, as shown in Fig. 3-A. The maximum bioprintable volume was determined by identifying the largest cylinder that could fit within the needle's range of motion without touching the FUGU's silicone walls. The compliance of the FUGU system at various volume, during the movement, was easily noticed. This flexibility is crucial for two reasons: (1) it allows for the movement of the printing needle within the system, and (2) it helps maintain the internal volume during the bioprinting process.

Through calculations, it was determined that the maximum bioprintable volume is 75 cm^3^ (forming a cylinder 4.5 cm in diameter and 4.7 cm high) when the FUGU is at its largest inflation (180 cm^3^). This volume represents 42% of the total FUGU volume. Interestingly, this efficiency ratio improves to 53% when using a 140 cm^3^ FUGU. These calculated bioprinted object volumes were not only theoretical but also practically validated through bioprinting experiments, as demonstrated in Fig. 3-B.

This analysis highlights the FUGU system's potential in bioprinting applications, highlighting its ability to adapt to different sizes and shapes while maintaining high efficiency and effectiveness in the bioprinting process.

**Figure 4:**
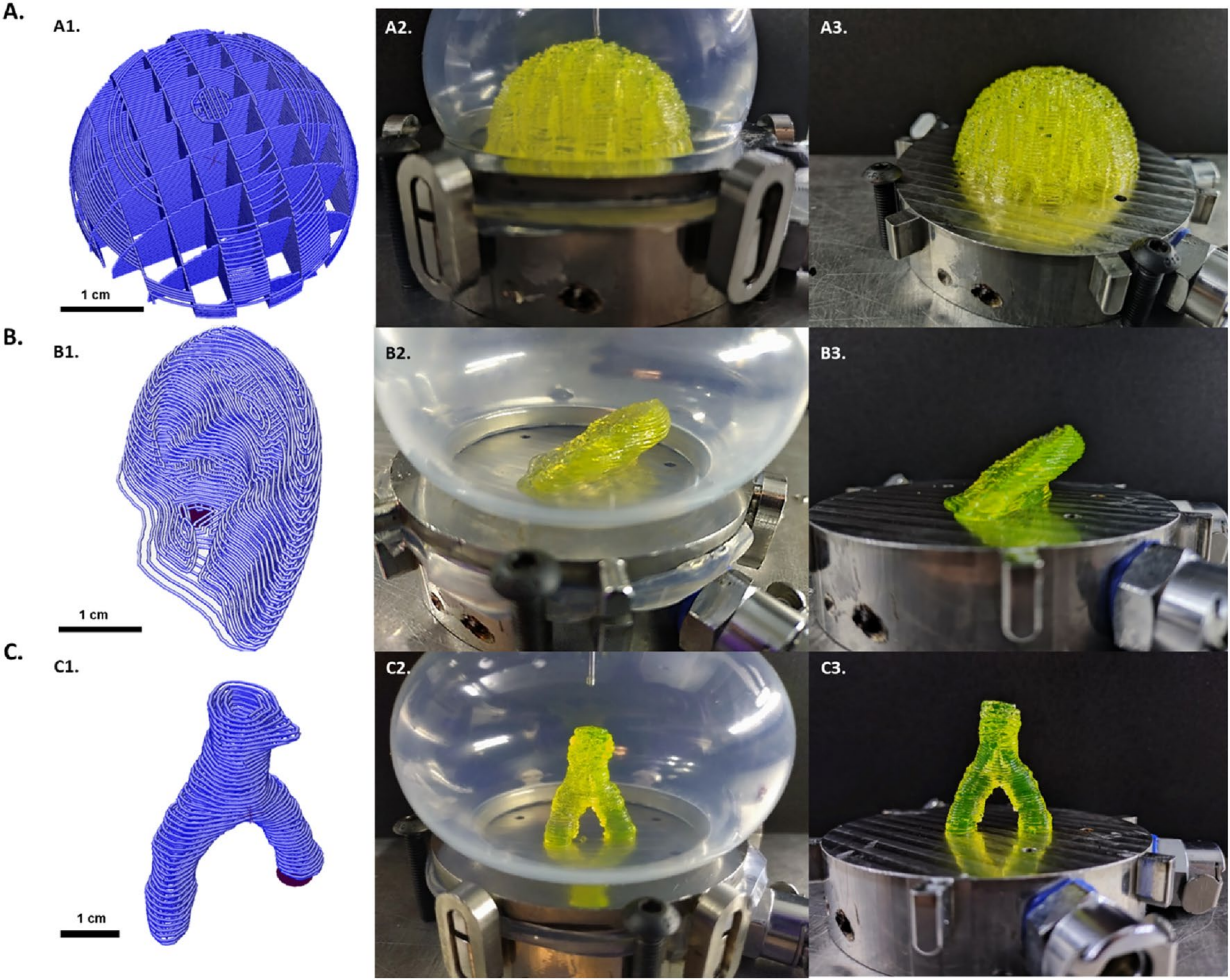
Versatility of the bioprinting in the FUGU device. (**A**) Bioprinting 33 cm^3^ 25% porosity hemisphere. A1/ Printing G-code visualisation. A2/ The bioprint within the FUGU. A3/ The bioprint once the FUGU membrane removed. (**B**) Bioprinting of a human ear shape. B1/ Printing G-code visualisation. A2/ The bioprint within the FUGU. A3/ The bioprint once the FUGU membrane removed. (**C**) Bioprinting of a 14 mm high vascular branching. C1/ Printing G-code visualisation. C2/ The bioprint within the FUGU. C3/ The bioprint once the FUGU membrane removed.

In order to evaluate the FUGU printing system versatility, 3 challenging shapes were printed using the DIY bioprinter: a 33 cm^3^ hemisphere (25% infill), a 4.5 cm long human ear and a 14 mm high vascular branching (Fig. 4). As can be seen, in all cases the compliance of the FUGU enabled the printing of the complex shapes without noticeable distortion. Videos of the printing can be found in Supplementary Video 1, 2 and 3.

To ensure compatibility across multiple bioprinting platforms, the FUGU system was designed to be compatible with a wide range of extrusion-based bioprinters. A crucial step in demonstrating this universality involved interfacing the FUGU with different bioprinters configurations. Indeed, each brands of bioprinter has its dedicated operating systems, which includes considerations such as motor torque and inner available space. To cover a large panel, we have chosen to work with the two most prevalent types of 3D bioprinter architectures: a Cartesian system^13^ and a 6-axis system^14^.

**Figure 5:**
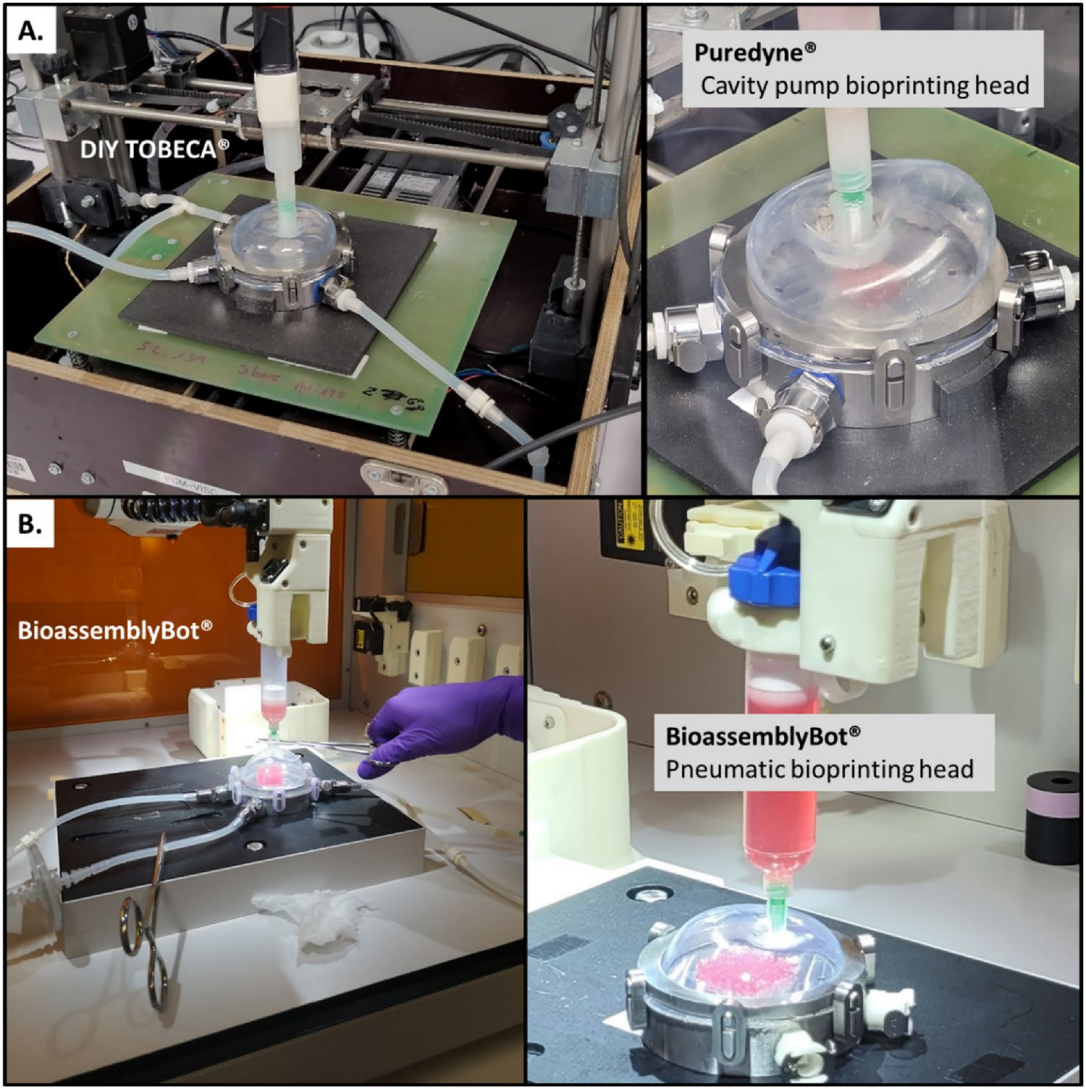
FUGU integration with micro-extrusion bioprinters. (**A**) Integration of the FUGU within a Do-It-Yourself (DIY) bioprinter from TOBECA®. The integration was performed using a Puredyne® cavity pump micro-extrusion system. (**B**) Integration of the FUGU with a 6-axis robotic bioprinter BioassemblyBot® (Advanced Solution LifeScience). The integration was performed using a pneumatic micro-extrusion system.

Identical FUGU systems in material composition, conception and size (85 cm^3^), were tested on these two types of bioprinters. The first was a DIY low-tech bioprinter (TOBECA®, France), and the second a sophisticated 6-axis robotic arm (Advanced Solutions Lifescience®, USA). In practice, the FUGU systems integrated seamlessly with both types of bioprinting setups (Fig. 5). Fundamental operations like needle insertion and deformation of the silicone membrane were executed smoothly, even with the relatively low torque (0.31 N.m) of the NEMA17 motor in the DIY bioprinter.

The versatility of the FUGU was also challenged with the two micro-extrusion systems available in the field, namely pneumatic and cavity pumps. Both systems were found to seamlessly interface with the FUGU (Fig. 4). This compatibility across various bioprinting platforms and micro-extrusion systems highlights the FUGU system's adaptability and potential for widespread application in the field of extrusion-based 3D bioprinting. Its design not only addresses the need for a universal bioprinting solution but also ensures ease of integration with existing technologies, paving the way for more innovative and flexible bioprinting approaches.

### Challenging the FUGU confinement

**Figure 6:**
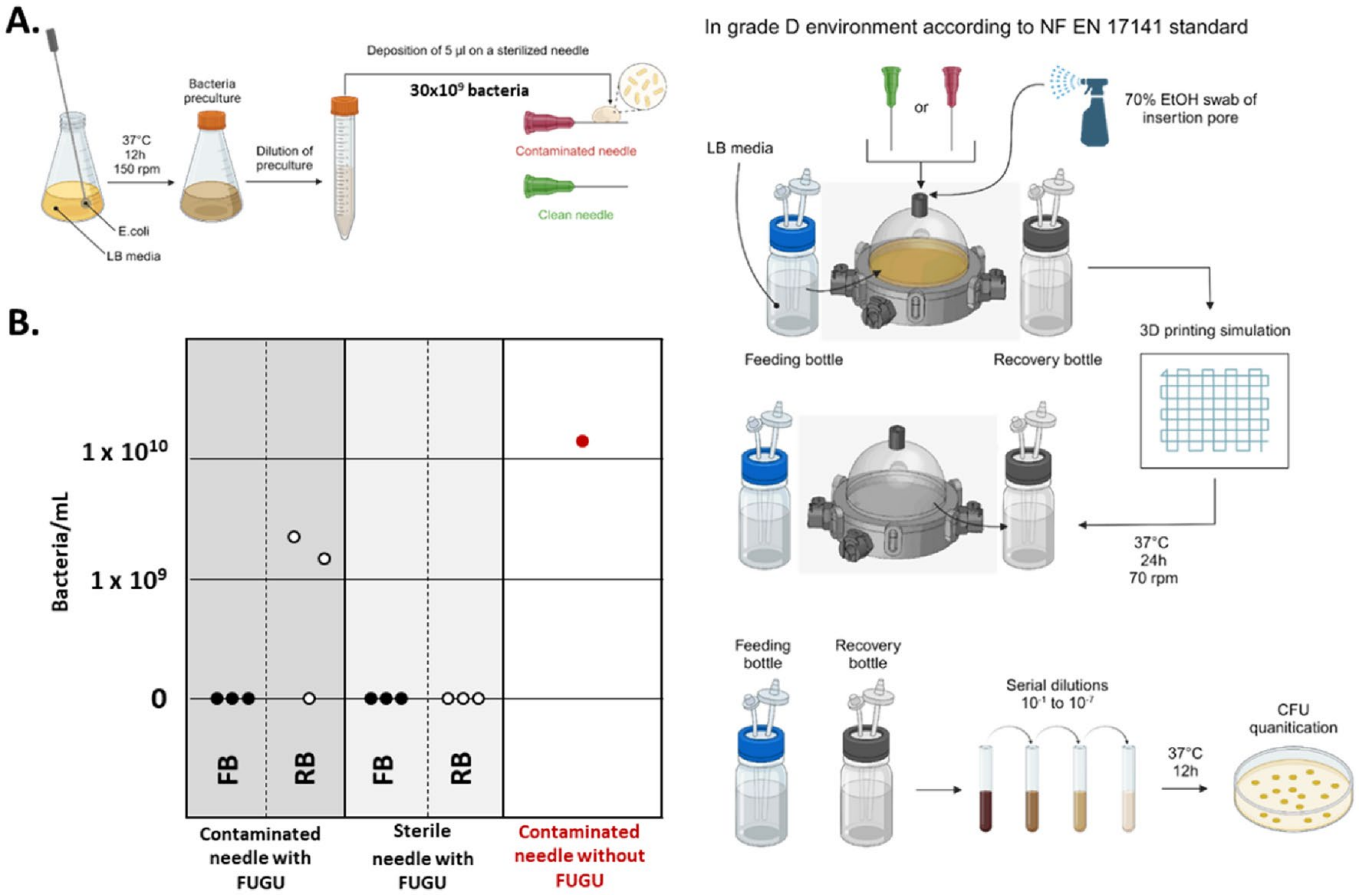
Evaluation of the sterile confinement within the FUGU. (**A**) Schematic representation of the logical steps involved in challenging confinement. (**B**) Numeration of the bacteria contamination through the needle insertion and bioprinting process.

The efficiency of the FUGU system's confinement was tested through sterility assays. The methodology for these tests is outlined in Fig. 6-A, involving the controlled introduction of bacterial charges. In addition to the introduction of bacteria, the FUGU was also challenged by performing the experiment in a grade D environment (NF EN 17,141) using the TOBECA® DIY bioprinter. The FUGU was filled with a bacterial culture medium, and the insertion pore was swabbed with ethanol. Then, bioprinting was simulated by inserting the needle through the insertion pore and immersing it in the bacterial culture medium. Bioprinting simulation lasted for 5 min, followed by complete needle removal.

Two different scenarios were assessed. The best case scenario was performing the experiment with an autoclaved 800 µm needle ensuring its complete decontamination before use. The worst case scenario was a deliberate and severe contamination of the needle with 30 × 10^9^ E. coli prior to its insertion through the pore.

Following bioprinting simulation, the culture medium inside the FUGU (Recovery Bottle RB) and remaining in the feeding Bottle (FB, acting as internal control) were incubated at 37 °C for 24 h. Culture media were plated on LB-agar solid medium for colony counting and incubated at 37 °C for 24 h. The results of grown bacteria enumeration are detailed in Fig. 6-B. Each aforementioned scenario was replicated three times.

In the best case scenario, despite the challenging conditions (no laminar flow, grade D environment, non-decontaminated DIY bioprinter), none of the three experiments resulted in contamination. This outcome demonstrates that even under strenuous conditions, simply swabbing the insertion pore with ethanol seems adequate to maintain sterility within the FUGU.

In the worst case scenario, two out of three experiments resulted in contamination within the FUGU. This indicates that even with a significantly high bioburden on the bioprinting needle, no detectable bacteria were transferred through the FUGU insertion pore in one case. This effect might be attributed to the elastomeric properties of the insertion pore, which could potentially clean the needle to some extent during its passage through the top and bottom diaphragms.

Overall, these results underscore the FUGU system's capability in maintaining sterility, highlighting its potential for safe and effective use in various bioprinting environments even under less-than-ideal conditions.

### Bioprinting and culturing within the FUGU

The FUGU system's capability for bioprinting and culturing large tissues was demonstrated through bioprinting a human tissue model and culturing for 16 Days. This model, measuring 14.5 cm^3^ with 30% porosity, comprised colorectal cancer cells (HT29) and cancer-associated fibroblasts (CAF). The bioprinted tissue was cultured statically within the FUGU system in a 37 °C/5% CO_2_ environment. Notably, during this period, no intervention such as culture medium renewal, or active gas perfusion were performed. The initial addition of 50 ml of culture medium to the 14.5 cm^3^ tissue containing 15.5 million cells was sufficient for the entire culture period. Furthermore, the gas exchange capabilities of the silicone membrane (i.e. the permeability for O_2_ and CO_2_ as described above) might have been sufficient to maintain appropriate pH and oxygen levels inside the FUGU.

**Figure 7:**
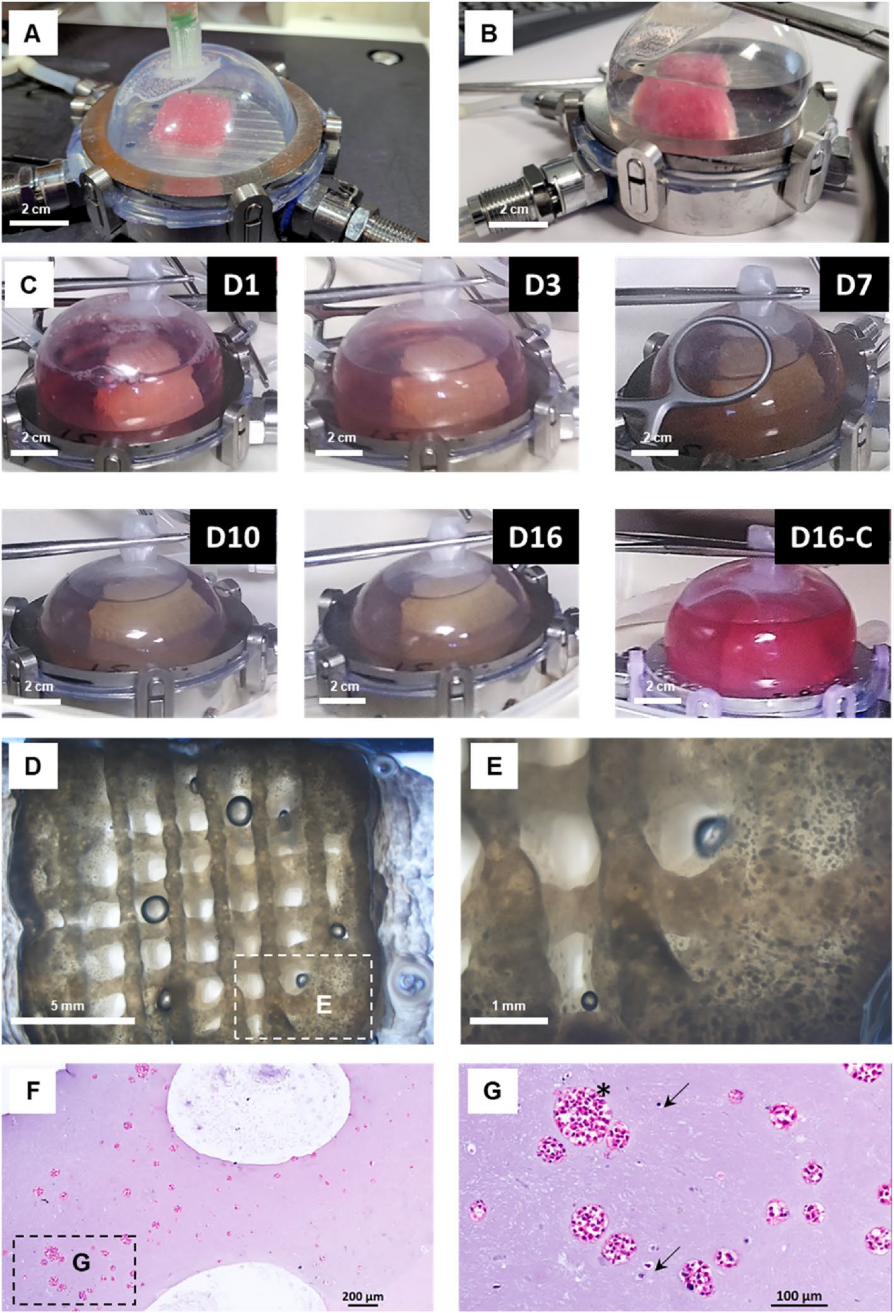
Bioprinting and culturing human tumour model within the FUGU. (**A**) Image of the FUGU during the bioprinting step. (**B**) Image of the FUGU during the consolidation step. (**C**) Images of the FUGU during 16 days of culture. D16-C corresponds to a control experiment without cells in the bioprinted object. (**D**) and (**E**) Contrast phase images of the tumour model at day 16. (**F**) and (**G**) Hematoxylin and eosin (**H**&**E**) stained histological sections of the tumour model at day 16.

Tissue production with the FUGU system (Fig. 7) begins with the bioprinting of a cell-laden hydrogel composed of gelatine, alginate, and fibrinogen (Fig. 7-A), followed by the consolidation of this hydrogel using calcium, thrombin, and transglutaminase (Fig. 7-B). The process then enters the culture phase.

The longitudinal monitoring of the culture, as depicted in Fig. 7-C, reveals significant changes in both the medium and tissue colour of cell-laden hydrogels over the 16-day maturation period, whereas no such changes were observed without cells. The tissue appeared to shrink and densify, indicative of cell growth and tissue remodelling, as suggested by previous studies^15^. The alteration in the colour of the culture medium further supported the hypothesis that the bioprinted tissue was actively producing pH-altering metabolites like lactate^16^.

Optical microscopy observations, shown in Figs. 7D,E, confirmed that after 16 days of confined culture, the bioprinted hydrogel was densely populated with HT29 spheroids^17,18^. These spheroids and the CAF cells were distinctly identified using H&E histology^19^, as demonstrated in Figs. 7F,G.

This experiment successfully demonstrates the FUGU system's effectiveness in facilitating the bioprinting and culturing of large-scale tissues under confined conditions, thus highlighting its potential for tissue engineering and regenerative medicine applications.

## Conclusions

The Flexible Unique Generator Unit (FUGU) system represents a significant advancement in 3D bioprinting and biofabrication. Its innovative design successfully addresses the critical issue of maintaining sterility throughout the bioprinting process, a challenge previously unmet by conventional methods. In contrast to bioprinters situated within a BSC or equipped with a built-in "sterilization" mechanism, where maintaining absolute sterility presents significant challenges due to continuous airflow and the potential introduction of contaminants from the surroundings, the FUGU (both stainless-steel and MAKROLON® polycarbonate) undergoes steam sterilization. This process ensures that its inner volume remains shielded from environmental contaminants throughout the bioprinting process.

The FUGU's unique combination of flexible silicone membranes and customisable parts, including a needle insertion pore and solid components made from medical-grade polycarbonate, showcases its adaptability and efficiency. However, scenarios such as repeated needle insertion and its impact on the permeability of the insertion pore were not addressed in this study and need further investigation.

The system's ability to adjust internal volumes and ensure sterility, even under non-ideal conditions, is a testament to its robust design. The testing in various environments, from custom-built basic cartesian to sophisticated 6-axis robotic arm bioprinters, demonstrates its compatibility, flexibility and universality across different bioprinting platforms. Nevertheless, certain aspects such as the force needed to deform the membrane without compromising printing accuracy could benefit from further refinement and understanding through Finite Element Method (FEM) analysis, an area that remains unexplored in this study.

The sterility assays under different infection scenarios highlight the system's effective confinement capabilities, physically protecting the tissues against contaminations. This feature is particularly critical in tissue engineering and regenerative medicine, where the risk of contamination can compromise the entire bioprinting process.

The FUGU's capacity to support bioprinting and culture of large tissues, as demonstrated with a human cancer tissue model, opens up new possibilities for creating complex tissue structures. The successful cultivation of a 14.5 cm^3^ tissue model, without external interventions, marks a significant milestone in the field.

Moreover, the system's gas permeability and the ability to allow optimal environmental conditions for cell growth and tissue development underscore its practicality and effectiveness. The observed tissue growth and remodelling during the 16-day culture period without medium changes or active gas exchange emphasise the FUGU's self-sufficiency and the efficiency of its design.

In summary, the FUGU system sets a new standard in 3D bioprinting technology, offering a versatile, efficient, and sterile environment for biofabrication. Its ability to maintain sterility, adapt to various bioprinting settings, and facilitate the growth and development of large-scale tissues positions it as an invaluable tool in advancing tissue engineering, regenerative medicine, and beyond. The FUGU system represents a technological breakthrough and a step towards the future of personalised medicine and complex tissue replacement therapies.

## Materials and methods

### Membrane and insertion pore production of the FUGU

Silicone 3D printing of the insertion pore was performed using an in-house developed silicone 3D printer equipped with a viper-HEAD 5 cavity pump (ViscoTec, Germany). To produce the insertion pore, a 30CC cartridge was filled with LSR4370 (ELKEM Silicones, France) formulated with 2% polyethylene glycol 400 (Sigma, France). The pore STL file was sliced using Simplify3D® software and printed using a 450 µm diameter tronconical nozzle (Nordson EFD, Germany). Once printed, the insertion pores were cured at 160 °C for 2 h before being used for FUGU membrane production.

The FUGU membranes were produced by moulding LSR4301 (ELKEM Silicones, France) on a Teflon surface. When the membranes comprised an insertion pore, the pore was dipped into the uncured LSR4301. For curing, the membranes were placed at 160 °C for 2 h.

### Building plate production

Stainless steel 316/316L building plates were produced by HUBS (Protolabs, France). Polycarbonate building plates were produced using Freeformer 300-3X (Arburg, Germany) and MAKROLON® pellets (Covestro, Germany). Optimized conditions for polycarbonate 3D printing were a printing head temperature of 285 °C and a printing chamber temperature of 185 °C, using a printing nozzle of 200 µm and a material discharge of 45%. The cross section of H2 traction samples were analysed through scanning electronic microscopy using a TM3000 (Hitachi, Japan). Mechanical properties of the 3D printed MAKROLON® were assessed in triplicate using uniaxial traction (Shimazu, Japan). Stainless steel valved connectors were used for fluid management (Colder Products Company, USA).

### Scanning of the FUGU and measurement of Z-distortion

The FUGU device was inflated with a low-pressure controller (Nordson EFD, Germany), and internal pressure was measured using a low-pressure manometer (RS component, France). 3D images of the inflated FUGU were obtained using a Space Spider 3D scanner (Artec 3D, Luxemburg). Acquisitions were used to determine FUGU volume after object reconstruction and segmentation (Artec Studio 18). Z-distortions were measured in triplicate using precision caliper.

### Gas permeation of the silicone membrane

Gas permeation experiments were performed by the *Laboratoire Polymères, Biopolymères et Surfaces*, UMR 6270 CNRS, Université de Rouen Normandie, France.

### Confinement testing

For confinement testing, qualified solutions of *Escherichia Coli* were prepared. Briefly, *E. Coli* stock solution (BL21 (DE3)) was amplified in Luria Bertani (LB) broth (Sigma, France) for 12 h at 37 °C under constant stirring at 150 rpm. This culture was normalized to McFarland 0.5 turbidity^20,21^ for the assay. Contaminated bioprinting needles were generated by depositing and drying 5 µL of this 0.5 turbidity *E. Coli* solution on the needle end for 10 min. The bacterial concentration was retrospectively estimated at 30 × 10^9^ bacteria/ml.

The needles, either autoclaved or contaminated, were used to simulate bioprinting for 5 min directly in LB broth, after being inserted through the insertion pore. Then, the LB broth from the inside of the FUGU was recovered and incubated for 24 h at 37 °C, as well as LB both remaining in the container used to fill the FUGU. These broth media were then serially diluted and plated on LB agar solid medium for subsequent colony counting.

### Printing complex shapes

Printing complex shapes (STL files available on demand) was performed using a specially formulated hydrogel composed of 28.5% (w/v) pluronic acid (F-127, Sigma, France) dissolved in Hanks’ Balanced Salt Solution (HBSS, Sigma, France). This gel was prepared at 4°C and loaded in 30CC cartridges before being connected to the printer and printed at room temperature. The different shapes were printed in the TOBECA® DIY bioprinter using a 800 µm diameter, 1.6 cm long needle (Nordson EFD, France).

### Bioprinting of human tissue

Human colorectal cancer tissues were produced by bioprinting HT29 colorectal cells (1.25 × 10^6^ cells/ml) (ATCC, HT29) and cancer-associated fibroblasts (0.375 × 10^6^ cells/ml) (NEUROMICS, CAF05). Just before printing, each cell type was trypsinized, suspended in fibrinogen and then formulated with gelatine and alginate to produce the cell-laden bioink as previously reported^22^. After homogenization, a 10 mL sterile cartridge (Nordson EFD, France) was filled with the cell-laden bioink, incubated for 15 min at 37 °C followed by 30 min at room temperature to stabilise the bioink rheological properties. The cartridge was loaded in a 6-axis robotic bioprinter (BioAssemblyBot®, Advanced Lifescience Solutions, USA) and used to print 14.5 cm^3^ (3 × 3 × 1.6 cm) tissue with 30% porosity. A 800 µm diameter, 1.6 cm long needle (Nordson EFD, France) was used to bioprint at a set speed of 10 mm/sec.

Bioprinted tissues were consolidated for 90 min at 37 °C in a solution containing 270 mM of CaCl_2_ (Sigma, France), 40 mg/mL of transglutaminase (Ajinomoto Activa WM, Japan) and 10 U/mL of thrombin (Sigma, France).

After the consolidation step, the FUGU containing the bioprinted tissues was filled with 50 mL of Roswell Park Memorial Institute (RPMI) medium (ATCC, France) supplemented with 10% calf serum (HyClone, USA), and placed at 37°C in a 5% CO_2_ incubator.

After a 16-day culture period without medium renewal, cancerous tissues were harvested and analysed through histological staining.

### Histological staining

Samples were rinsed in 1 × phosphate buffered saline (PBS) solution and fixed inparaformaldehyde (Antigenfix solution, DiaPath, France) overnight at 4 °C. They were then rinsed in PBS, followed by dehydration in a gradient of increasing ethanol concentrations from 30 to 70% and stored at 4 °C in 70% ethanol until paraffin embedding. For paraffin embedding, the samples were first incubated in 90% ethanol at 35 °C followed by successive incubations in two 100% ethanol baths at 55 °C. The samples were then transferred successively to two methylcyclohexane baths at 55 °C. Each incubation lasted for 10–15 min. Then, the samples were incubated in paraffin for 28 min at 60 °C for paraffin impregnation. It was followed by a second incubation in fresh paraffin at 60 °C for 5 min. Samples were sliced down to 5 µm thin sections. For Hematoxylin–Eosin (HE) staining, sections were deparaffinized and rehydrated by successive incubation in toluene, followed by decreasing gradients of ethanol from 100 to 70% and finally water. The Hematoxylin Eosin Fast Quick kit (Diapath, France) was used to stain the sections.

### Supplementary Information

Supplementary Information 1.

Supplementary Video 1.

Supplementary Video 2.

Supplementary Video 3.

## Data Availability

The following data were generated during the study to produce the different figures: - Figure 1: CAD file of the FUGU system designed using SOLIDWORKS. - Figure 2: Inflation 3D files of the FUGU generated using ARTEC studio. Deflation raw measurement data. Full set of electronic microscopy images. Raw data of traction test. - Figure 3: Full set of images used for deformation tracking. - Figure 4: Full Gcode of the 3D bioprinting step. - Figure 6: Full set of colony counting images. - Figure 7: Full set of histology images. All these datasets used and/or analysed during the current study are available from the corresponding author on reasonable request.

## References

[1] Mota C, Camarero-Espinosa S, Baker MB, Wieringa P, Moroni L (2020). Bioprinting: From tissue and organ development to in vitro models. Chem. Rev..

[2] Mladenovska T, Choong PF, Wallace GG, O'Connell CD (2023). The regulatory challenge of 3D bioprinting. Regen. Med..

[3] Mao H (2020). Recent advances and challenges in materials for 3D bioprinting. Progr. Natl. Sci.: Mater. Int..

[4] Tong A (2021). Review of low-cost 3D Bioprinters: State of the market and observed future trends. SLAS Technol..

[5] Prevention, C. C. F. D. C. A. Glossary of Terms for Infection Prevention and Control in Dental Settings, https://www.cdc.gov/oralhealth/infectioncontrol/glossary.htm (2020).

[6] Newsom SW, Walsingham BM (1974). Sterilization of the biological safety cabinet. J. Clin. Pathol..

[7] Rosser, J. & Thomas, D. J. in *3D Bioprinting for Reconstructive Surgery* (eds Daniel J. Thomas, Zita M. Jessop, & Iain S. Whitaker) 191–215 (Woodhead Publishing, 2018).

[8] Thangadurai M, Srinivasan SS, Sekar MP, Sethuraman S, Sundaramurthi D (2024). Emerging perspectives on 3D printed bioreactors for clinical translation of engineered and bioprinted tissue constructs. J. Mater. Chem. B.

[9] Sarkar N, Bhumiratana S, Geris L, Papantoniou I, Grayson WL (2023). Bioreactors for engineering patient-specific tissue grafts. Nat. Rev. Bioeng..

[10] Lamberti A, Marasso SL, Cocuzza M (2014). PDMS membranes with tunable gas permeability for microfluidic applications. RSC Adv..

[11] Junne S, Neubauer P (2018). How scalable and suitable are single-use bioreactors?. Curr. Opin. Biotechnol..

[12] Courtial EJ (2019). Silicone rheological behavior modification for 3D printing: Evaluation of yield stress impact on printed object properties. Addit. Manuf..

[13] Tong A (2021). Review of low-cost 3D Bioprinters: State of the market and observed future trends. SLAS Technol..

[14] Li K (2023). Advancements in robotic arm-based 3D bioprinting for biomedical applications. Life Med..

[15] Chastagnier L (2023). Deciphering dermal fibroblast behavior in 3D bioprinted dermis constructs. Bioprinting.

[16] Ikari R (2021). Differences in the central energy metabolism of cancer cells between conventional 2D and novel 3D culture systems. Int. J. Mol. Sci..

[17] Cadamuro F (2023). 3D bioprinted colorectal cancer models based on hyaluronic acid and signalling glycans. Carbohydr. Polym..

[18] Magdeldin T (2014). The efficacy of cetuximab in a tissue-engineered three-dimensional in vitro model of colorectal cancer. J. Tissue Eng..

[19] McGuckin C (2023). World’s first long-term colorectal cancer model by 3D bioprinting as a mechanism for screening oncolytic viruses. Cancers.

[20] in *Clinical Microbiology Procedures Handbook* 5.20.21.21–25.20.23.10 (2016).

[21] in *Clinical Microbiology Procedures Handbook* 5.2.1.1–5.2.2.10 (2016).

[22] Pourchet LJ (2017). Human skin 3D bioprinting using scaffold-free approach. Adv. Healthc. Mater..

