# Supplementary material for: Confined bioprinting and culture in inflatable bioreactor for the sterile bioproduction of tissues and organs
Source: Sci Rep. 2024 May 14;14:11003. doi: 10.1038/s41598-024-60382-2 (PMC11093974; doi:10.1038/s41598-024-60382-2)
Supplement: Supplementary file 1 — Supplementary Information 1. [file 41598_2024_60382_MOESM1_ESM.docx]

Confined bioprinting and culture in inflatable bioreactor for the sterile bioproduction of tissues and organs

Alexandre DUFOUR^1^, Lucie Essayan^1^, Céline THOMANN^1^, Emma PETIOT ^1^, Isabelle GAY ^2^, Magali BARBAROUX ^2^, Christophe MARQUETTE ^1^

*1 3d.FAB, CNRS, INSA, CPE-Lyon, UMR5246, ICBMS, Universite Claude Bernard Lyon 1, Villeurbanne, France*

*^2^Sartorius Stedim FMT, Aubagne, France*

**Supplementary Video title and legend**

***Supplementary video 1:*** *Porous hemisphere bioprinting within the FUGU confined system.*

***Supplementary video 2:*** *Vascular branching shape bioprinting within the FUGU confined system.*

***Supplementary video 3:*** *Human ear shape bioprinting within the FUGU confined system.*
